# Benefit finding and post-traumatic growth in long-term colorectal cancer survivors: prevalence, determinants, and associations with quality of life

**DOI:** 10.1038/bjc.2011.335

**Published:** 2011-08-30

**Authors:** L Jansen, M Hoffmeister, J Chang-Claude, H Brenner, V Arndt

**Affiliations:** 1 Division of Clinical Epidemiology and Aging Research, German Cancer Research Center (DKFZ), POB 10 19 49, 69009 Heidelberg, Germany; 2 Division of Cancer Epidemiology, German Cancer Research Center (DKFZ), Heidelberg, Germany

**Keywords:** benefit finding, colorectal cancer, long-term, post-traumatic growth, quality of life, cancer survivors

## Abstract

**Background:**

As research on quality of life of colorectal cancer (CRC) survivors has mainly focused on downsides of cancer survivorship, the aim of this study is to investigate benefit finding (BF) and post-traumatic growth (PTG) in long-term CRC survivors.

**Methods:**

Benefit finding, PTG, and quality of life were assessed 5 years after diagnosis in a population-based cohort of 483 CRC patients using the benefit finding scale, the post-traumatic growth inventory, and the EORTC QLQ-C30. Prevalence of BF and PTG, determinants of moderate-to-high BF and PTG, and the association between BF, PTG, and quality of life were investigated.

**Results:**

Moderate to high levels of BF and PTG were experienced by 64% and 46% of the survivors, respectively. Survivors with the highest level of education and with higher depression scores reported less BF and PTG. The PTG increased with increasing stage and self-reported burden of diagnosis. Quality of life only correlated weakly with PTG (Pearson's *r*=0.1180, *P*=0.0112) and not with BF (*r*=0.0537, *P*=0.2456).

**Conclusion:**

Many long-term CRC survivors experience BF and PTG. As these constructs were not strongly correlated with quality of life, focusing solely on quality of life after cancer misses an important aspect of survivorship.

Colorectal cancer (CRC) is one of the most common malignant diseases worldwide. As prognosis of CRC has improved over the last years (Verdecchia *et al*, 2007), research on quality of life of CRC survivors has become increasingly important. This research has mainly focused on the negative experiences of cancer survivors like long-term symptoms and restrictions in quality of life (Jansen *et al*, 2010). But studies have shown that a high percentage of cancer survivors also report positive changes in the context of their disease (Dunn *et al*, 2006; Salsman *et al*, 2009; Rinaldis *et al*, 2010). Two constructs of positive consequences of cancer have been distinguished: post-traumatic growth (PTG) and benefit finding (BF). PTG refers to benefits associated with changes in life perspective, interpersonal relationship, and self-perception. The changes result from the struggle of an extreme event like a cancer diagnosis and treatment and cannot be caused by minor stressors (Tedeschi and Calhoun, 1995; Sumalla *et al*, 2009) . In contrast, BF is defined as the process in which the patient re-assigns positive value to the illness based on the benefits he or she identifies (Collins *et al*, 1990; Helgeson *et al*, 2006). While BF is hypothesised to start immediately after diagnosis, PTG refers to an active change in one's capacity to deal with adverse events and, thus, may develop even years after the cancer diagnosis (Calhoun and Tedeschi, 1998). Despite their distinct definitions, these terms have often been used interchangeably (Lechner *et al*, 2003; Mols *et al*, 2009; Sumalla *et al*, 2009).

Most previous studies on PTG and BF were based on samples including breast cancer or mixed cancer sites with assessments of PTG and BF in the first 2–3 years after diagnosis. Results from these studies have shown that around 80% of the survivors regard themselves as having benefited in some way from their cancer experience (Sumalla *et al*, 2009). Benefits were reported among others for life satisfaction/appreciation, relationship with others, and personal strength. Results on levels and determinants of BF and PTG were shown to be disease specific (Barskova and Oesterreich, 2009) and, thus, cannot be directly transferred to survivors of other cancers. Few studies investigated BF and PTG in CRC survivors (Dunn *et al*, 2006; Salsman *et al*, 2009; Rinaldis *et al*, 2010). However, these studies included short-term survivors only. To get further insight into PTG and BF in long-term CRC survivors, the aim of this paper is to investigate the prevalence of BF and PTG and to determine what socio-demographic, clinical, and psycho-social factors distinguish CRC patients who show a high level of BF and PTG from those who experience only low levels.

## Materials and methods

### Study design and study population

This analysis includes patients with CRC from a population-based case–control study (DACHS study) carried out in southwest Germany (Rhine-Neckar-Odenwald and Heilbronn Region). Further details about baseline recruitment in this study have been described elsewhere (Hoffmeister *et al*, 2009; Brenner *et al*, 2011).

In brief, patients who were mentally and physically able to participate in a personal interview of about 1 h were recruited by their clinicians, usually a few days after surgery. After an average of 3 years since diagnosis, detailed information on CRC treatment was collected from oncologists and other attending physicians in a standardised questionnaire. In 2009, a 5-year follow-up was conducted with 906 patients diagnosed in 2003 or 2004. After ascertainment of vital status and dates of death through population registries and after exclusion of patients who moved abroad (*N*=1) or with unknown new mailing address (*N*=1), a questionnaire was sent to all patients alive (*N*=585) who had not denied further contacts (*N*=584). Non-responders were mailed up to two reminder letters, which were followed by one reminder phone call in case of non-response to mailings. If patients rejected to return the questionnaire, a phone interview on the basis of a shortened version of the questionnaire (referred to as short questionnaire; details see below) was offered. Both questionnaires entailed questions concerning new concomitant diseases or recurrences of CRC. In case of reported new diagnoses including cancer recurrence, attending physicians were contacted to validate the diagnoses. Physicians were also contacted when information on CRC recurrence within 5 years after diagnosis was lacking or uncertain.

All participants gave written informed consent. The study was approved by the ethics committees of the University of Heidelberg and the state medical boards of Baden-Wuerttemberg and Rhineland-Palatinate.

### Follow-up questionnaire

Benefit finding was assessed with the short form of the benefit finding scale (BFS; Antoni *et al*, 2001; Mohamed and Böhmer, 2004). The BFS has 10 items on a five-point Likert scale (Table 1) ranging from 1 (not at all) to 5 (extremely), which are summarised in four subscales (acceptance, sensitivity to others, improved coping, and new purpose of life) and a total BF. The PTG was measured by three scales (appreciation of life, new possibilities, and spiritual change) of the PTG inventory (Tedeschi and Calhoun, 1996; PTGI, Maercker and Langner, 2001). The PTGI scale ‘personal strength’ was not included, as the factor structure was not replicable in the German version (Maercker and Langner, 2001). The scale ‘relationship to others’ was excluded to avoid redundancies with other instruments and to reduce the overall length of the questionnaire. The implemented shortened version of the PTG entailed 10 items on a six-point Likert scale (Table 1) ranging from 0 (not at all) to 5 (to a very great degree). Quality of life was assessed with the Quality of Life Questionnaire Core 30 Items (QLQ-C30; Aaronson *et al*, 1993) developed by the European Organisation for Research and Treatment of Cancer (EORTC). Psychological distress was assessed with the short form of the ‘questionnaire on stress in cancer patients revised version’ (QSC-R10; Herschbach *et al*, 2004). The 15-item short form of the geriatric depression scale (GDS) was included to assess depression (Yesavage *et al*, 1982; Gauggel and Birkner, 1999), as it was specifically developed for older persons and has been validated in cancer patients as well as older persons (Nelson *et al*, 2010). Additionally, the questionnaire included items to assess the identification as cancer patients (‘Do you still consider yourself as a cancer patient?’) and the self-reported burden of diagnosis and burden of treatment (‘How stressful were the following aspects of your colorectal cancer disease and treatment?’ – ‘initial diagnosis’, ‘treatment (chemotherapy, radiotherapy, and surgery)’).

A short questionnaire, which was used in case a patient was not willing to complete the full questionnaire, contained the global QLQ-C30 item as sole quality of life measure and some single questions on other domains but not on BF or PTG.

### Statistical methods

The distribution of patient and disease characteristics was compared between responders, short-questionnaire responders, and non-responders to assess the potential impact of non-random missing data.

The scoring of the QLQ-C30, BFS, QSC-R10, and GDS was performed according to the scoring manuals. In case of missing items in the BFS or the QLQ-C30, multi-item scores were calculated as the mean of non-missing items if at least half of the items from the corresponding scale had been completed. The PTGI summary scores are usually computed as the sum of the item scores. In accordance with the BFS scoring, we calculated the mean of the PTGI item scores in order to handle missing items identically. In sensitivity analysis, we repeated the analyses using the sum instead of the mean of the item scores and the results were comparable.

Cutoff values defining the prevalence of BF or PTG (BF/PTG) were determined *a priori* (Table 1). In general, lower scores represent lower levels of BF and PTG. Prevalence of any BF/PTG was defined as checking more than the lowest item score (‘not at all’) at least once. Prevalence of moderate-to-high BF/PTG was defined by a mean score ⩾3, respectively.

Univariate logistic regression models were computed to estimate the association between the prevalence of moderate to high total BF/PTG and *a priori* defined potential determinants of BT/PTG:

- Socio-demographic factors (sex, age at follow-up, partnership status at follow-up, highest school degree at baseline, number of real friends/relatives/family members at baseline).
- Clinical factors (comorbidity at baseline, tumour stage, amount of adjuvant/neoadjuvant therapy (none, radiotherapy, chemotherapy, chemotherapy, and radiotherapy), CRC recurrence previous to follow-up).
- Psycho-social factors (self-reported burden of diagnosis, self-reported burden of treatment, identification as cancer patient, psychological distress of cancer patients (QSC-R10), and depression score (GDS)).

In addition, multiple logistic regression models were computed to determine the independent association between each of the aforementioned factors and BF/PTG while controlling for all socio-demographic and clinical factors. We did not adjust for psycho-social factors to estimate the effect of clinical and socio-demographic factors independent of potential interdependencies of the outcomes with psycho-social factors. The analysis was repeated without dichotomising the BF and PTG scores using analysis of covariance.

The association between BF, PTG, and quality of life was estimated by Pearson's correlation coefficients. Furthermore, the mean functioning and global quality of life scores of survivors with moderate-to-high BF/PTG were compared with survivors with lower BF/PTG using a *t*-test and a multiple linear regression with adjustment for all factors that were significant in the analysis of determinants.

All analyses were performed with SAS software, version 9.2 (SAS Institute Inc., Cary, NC, USA). Statistical significance was defined by a two-sided *P*<0.05. No multiple comparison corrections were made given the exploratory nature of the analysis.

## Results

Of the 584 survivors in the 5-year follow-up, 23 were excluded from the survey, as they were physically or mentally not able to participate (e.g., because of dementia or hospitalisation), 19 did not respond to mailings and could not be contacted by phone, 12 agreed to participate, but did not return the questionnaire, and 21 actively refused to participate. The remaining patients returned a full (*N*=483) or short (*N*=25) questionnaire resulting in a response rate of 86% (full questionnaire) and 91% (full and short questionnaires), respectively.

Patient characteristics according to response status are shown in Table 2. At 5-year follow-up, mean age of survivors who returned a full questionnaire (full responders) was 72 years (standard deviation: 9 years). Of these survivors, 38% were female, 69% had completed 9 or fewer years of education, and 76% were living with a partner. The average time since diagnosis was 5.4 years (standard deviation: 0.4, range: 4.8–6.4 years). About 97% of the full responders had CRC stage I, II, or III at baseline (in almost equal shares), whereas only 3% had stage IV. The tumour had been located in the colon for 59% of these survivors, 98% had received surgery, and 58% had received any adjuvant or neoadjuvant treatment. A recurrence of CRC had occurred in 7%. Compared with non-responders including short-questionnaire responders, who were not included in the analysis, full responders were on average younger, more often male, had more often rectal cancer, and received more often adjuvant and/or neoadjuvant treatment. Overall quality of life was lower for short-questionnaire responders than for full responders, but the difference was not statistically significant (*Δ*=7.62, *P*=0.1209).

### Prevalence of BF and PTG

Almost all survivors experienced BF and PTG at least to some degree (Table 3) and more than half of the survivors reported moderate to high levels of total BF (64%) and of the subscales acceptance (62%), sensitivity to others (57%), improved coping (56%), and new purpose of live (53%). Moderate to high levels of PTG were reported by 46% of the survivors. While 70% of the survivors experienced moderate to high levels of appreciation of life, only 33% and 30% reported moderate to high levels for the subscales spiritual change and new possibilities.

### Determinants of BF and PTG

The odds ratios (ORs) of the association between the potential determinants and the prevalence of moderate-to-high BF/PTG are presented in Table 4. Of the socio-demographic factors, only level of education was significantly associated with the prevalence of moderate-to-high BF. Survivors with higher education reported less often moderate to high levels of BF (after adjustment: *P*_Trend_=0.0088). For PTG, a similar but non-significant pattern was observed. Again, no other socio-demographic variable was significantly associated with moderate-to-high PTG, but older survivors tended to report less often PTG.

In the univariate analysis of clinical and psycho-social factors, the chance of experiencing moderate-to-high PTG increased significantly with the stage at diagnosis, the provision of chemotherapy, and the self-reported burden of diagnosis (Table 4). A similar but non-significant pattern was observed for the self-reported burden of treatment. After adjustment, only the association between burden of diagnosis and prevalence of moderate-to-high PTG remained significant (*P*_Trend_=0.0028). For BF, only the comparison between survivors reporting modest compared with high burden of diagnosis was significant. After adjustment, the chance to experience moderate-to-high BF was 41% lower for survivors reporting modest burden of diagnosis.

The distress level and the identification as a cancer patient 5 years after diagnosis were not associated with BF or PTG. The prevalence of high-to-moderate BF and PTG significantly decreased with the depression score 5 years after diagnosis (after adjustment, for BF: OR 0.91 (0.86–0.97), for PTG: OR 0.92 (0.86–0.98)).

Further in-depth analysis of the determinants of BF and PTG, where we used the original score as dependent variable, did not materially change the observed findings derived from using dichotomised PTG and BF scores (data not shown).

### Association between BF, PTG, and quality of life

Total PTG and total BF were strongly correlated (Pearson's *r*=0.71). The correlation between quality of life and BF/PTG is shown in Table 5. Total BF and the domains of BF were not significantly correlated with global quality of life or any functioning score. Total PTG and the PTG domain ‘appreciation’ of life were significantly positively associated with global quality of life and physical functioning, but the correlations were small (all *r*<0.16). The PTG domain ‘new possibilities’ was significantly but weakly correlated with global quality of life (*r*=0.13), physical (*r*=0.13), and role functioning (*r*=0.10). Table 6 shows the mean quality of life scores according to the total PTG and BF levels. Only global quality of life was significantly associated with PTG. Mean global quality of life was 4.5 points lower for survivors who experienced lower PTG.

## Discussion

To our knowledge, this is the first population-based study examining BF and PTG in long-term CRC survivors. Five years after diagnosis, almost all CRC survivors experience BF or PTG at least to some degree with about half of them reporting moderate to high levels of BF and PTG. Domains with the highest prevalence of moderate to high growth were appreciation of life and acceptance.

Analysing determinants of BF and PTG may help to better understand these constructs and the factors that may influence the adjustment of cancer survivors to their disease. In our study, education was the only socio-demographic variable that was significantly associated with BF and PTG. In accordance with previous studies including short-term CRC survivors and mixed cancer samples (Widows *et al*, 2005; Jaarsma *et al*, 2006; Rinaldis *et al*, 2010), respectively, survivors with a higher level of education experienced less BF and PTG. Interpreting lower education as a proxy of lower socioeconomic status, one explanation for this finding may be that survivors with a lower socioeconomic status may routinely be confronted with hardships in their lives and, thus, may be more experienced in finding something positive from negative events (Tomich and Helgeson, 2004; Rinaldis *et al*, 2010).

In contrast to our study, which did not show a significant association between BF/PTG and age, studies that included survivors with various cancer sites reported that younger survivors experience more BF/PTG than older survivors (Gotay and Muraoka, 1998; Lechner *et al*, 2003; Morris *et al*, 2007). For long-term breast cancer survivors, one study on BF/PTG found a significant association between age and the PTG domain ‘new possibilities’ only (Mols *et al*, 2009), whereas another study on PTG did not find any significant association between age and PTG (Lelorain *et al*, 2010). The lack of a strong association between BF/PTG and age in our study might reflect the presumably narrower age range of our study that included CRC survivors only as compared with studies encompassing survivors with various cancer sites. Likewise, it might reflect an attenuation of age-specific differences over the long run as previous studies that reported consistent age effects included short-term survivors only.

Post-traumatic growth is thought to develop as a consequence of the struggle from the cancer diagnosis and treatment. Thus, the amount of perceived growth was hypothesised to be associated with the real or perceived life threat of the traumatic event (Tedeschi and Calhoun, 2004). In our study, the prevalence of moderate-to-high PTG was higher for survivors who had a higher objective burden of their disease, such as a higher stage at diagnosis or higher intensity of therapy, or a higher self-reported burden of diagnosis. In accordance with our results, previous studies in other cancer sites showed associations with perceived life threat (Lechner *et al*, 2003; Sears *et al*, 2003; Lelorain *et al*, 2010), severity of disease (Tomich and Helgeson, 2004; Urcuyo *et al*, 2005), and chemotherapy (Bower *et al*, 2005; Lelorain *et al*, 2010). For BF, we did not find such a consistent association with burden of disease. It has been reported that the association between BF and stage may be curvilinear, as cancer survivors with stage II had higher BF than survivors with stage I and IV (Lechner *et al*, 2003), but our results do not support this pattern.

While psychological distress during follow-up was not associated with BF or PTG, the prevalence of moderate-to-high BF and PTG was lower for survivors with higher depression scores at follow-up. Results from other studies on the association between depression and BF (Antoni *et al*, 2001; Urcuyo *et al*, 2005) and PTG (Cordova *et al*, 2001; Salsman *et al*, 2009) including breast cancer or short-term CRC survivors are inconsistent.

Overall, determinants of BF and PTG were found to be different, especially with respect to the association with stage, therapy, and burden of disease. Nonetheless, BF measured by the BFS and PTG measured by three subscales of the PTGI were strongly correlated. This result suggests that BF and PTG are related but independent constructs and the terms should be clearly defined and not used interchangeably.

In accordance with results from studies on other cancer sites (Cordova *et al*, 2001; Tomich and Helgeson, 2002; Mols *et al*, 2009), BF and PTG did not correlate with quality of life in long-term CRC survivors in our study. Survivors who experienced moderate-to-high PTG reported significantly higher mean global quality of life. However, the marginal size of the effect is unlikely to be clinically relevant (Osoba *et al*, 1998). We also did not find any non-linear associations, as was previously reported by Lechner *et al* (2006). As the EORTC QLQ-C30 measures specifically health-related quality of life, we additionally computed the correlation between BF/PTG and the single item on overall quality of life included in our questionnaire. The results were comparable to the result on the domain ‘quality of life’, which includes this single item together with an item on overall health. Thus, we did not find a meaningful association between BF/PTG and overall quality of life either. Due to this independence of quality of life and BF/PTG, the generally reported high global quality of life of long-term CRC survivors (Jansen *et al*, 2010) cannot be explained by the experience of BF and PTG. In addition, both positive and negative consequences of cancer survivorship must be investigated to get a comprehensive understanding of the adjustment of cancer patients to their disease.

A caveat to be considered in the interpretation of our study is that the PTGI was not directly developed to assess PTG in CRC survivors. Also, validation studies were based on college students (Tedeschi and Calhoun, 1996) and breast cancer patient samples (Brunet *et al*, 2010). The BFS was developed to assess BF in breast cancer patients (Antoni *et al*, 2001), but the German version was validated on a mixed cancer sample including mainly CRC patients (Mohamed and Böhmer, 2004). Thus, the BFS factor structure has been replicated in this survivor group. But due to the general focus on other cancer sites, the instruments assessing BF and PTG may miss important domains of growth that specifically arise for CRC patients, which are on average older and more often male. However, the high prevalence of BF and PTG in our study sample suggests that the assessed domains also apply to long-term CRC survivors.

A further caveat of the study is the restriction to three of the five domains of PTG. We decided to exclude the PTGI scale ‘personal strength’, as the factor structure was not replicable in the German version (Maercker and Langner, 2001). To avoid redundancies and to restrict the length of the questionnaire, the scale ‘relationship to others’ was additionally excluded. As a consequence, our assessment may miss some specific aspects of PTG. In addition, our estimates for total PTG may not be comparable to estimates from other studies that included the complete PTGI.

Strengths of our study are the population-based design, the high response rate, and the concurrent assessment of negative and positive consequences of disease and treatment with validated instruments.

In conclusion, our results show that BF and PTG are highly prevalent among long-term CRC survivors. Thus, to get a comprehensive understanding of the adjustment of cancer patients after diagnosis, negative as well as positive consequences of cancer survivorship need to be investigated. Quality of life was only weakly related to BF and PTG and, thus, the generally reported high global long-term quality of life after cancer cannot be directly explained by positive adjustments.

## Figures and Tables

**Table 1:** Dichotomisation of the response levels of the BFS and PTGI

**BFS**		**PTGI**		
**Response levels**	**Score**	**Response levels**	**Score**	**Dichotomisation**
Not at all	1	Not at all	0	No BF/PTG or low levels of BF and PTG
A little	2	Very small degree	1	
		Small degree	2	
				
Moderately	3	Moderate	3	Moderate to high level of BF and PTG
Quite a bit	4	Great degree	4	
Extremely	5	Very great degree	5	

Abbreviations: BFS=benefit finding scale; PTGI=post-traumatic growth inventory; BF=benefit finding; PTG=post-traumatic growth.

**Table 2:** Characteristics of study population according to response status

			**According to responder status in 5-year follow-up**			
	**Baseline (*N*=906)**	**Deceased (*N*=320)**	**Full responder (*N*=483)**	**Short-questionnaire responder (*N*=25)**	**Non-responder (*N*=78)**	***χ*^2^-test: (*P*) responder *vs* Non-responder^a^**
*Sex*						**0.0072**
Female	42%	45%	38%	60%	50%	
Male	58%	55%	62%	40%	50%	
						
*Age at follow-up (years)*						**<0.0001**
−59	9%	7%	11%	0%	12%	
60–69	23%	18%	28%	20%	15%	
70–79	37%	33%	42%	24%	24%	
80+	31%	43%	20%	56%	49%	
Mean (s.d.)	73.7 (10.2) years	76.2 (10.5) years	71.3 (9.2) years	79.6 (8.5) years	76.2 (11.3) years	
Living with partner at follow-up	NA	NA	76%	56%	NA	NA
						
*School education*						0.1556
⩽9 years	70%	71%	69%	96%	72%	
10–11 years	16%	16%	17%	0%	14%	
12+ years	14%	14%	14%	4%	14%	
						
*Location*						**0.0057**
Colon	63%	65%	59%	84%	71%	
Rectum	37%	35%	41%	16%	30%	
						
*Stage*						0.0861
I	24%	8%	33%	36%	27%	
II	32%	23%	35%	52%	46%	
III	30%	32%	30%	8%	27%	
IV	14%	36%	3%	4%	0%	
Surgery	98%	98%	98%	92%	96%	0.1043
						
*Adjuvant/neoadjuvant therapy*						**0.0095**
No therapy	54%	41%	58%	80%	68%	
Radiotherapy	2%	1%	3%	4%	5%	
Chemotherapy	30%	42%	24%	16%	20%	
Radio- and chemotherapy	14%	16%	15%	0%	7%	
						
*Colorectal cancer recurrence*						0.6694
Yes	19%	41%	7%	8%	8%	
No	80%	59%	93%	92%	81%	
Missing	1%	0%	0%	0%	12%	

Abbreviation: s.d.=standard deviation. Among the variables included in the analysis, the proportion of missing values was <5% except for burden of treatment (5%), stress (11%), and depression score (6%). In general, older survivors, female survivors, survivors without a partner, and survivors with stage I compared with stage III and IV were more reluctant to provide information on these items. Significant *P*-values (*P*<0.05) are highlighted in bold.

a Short-questionnaire responders are counted as non-responders.

**Table 3:** Mean (s.d.) and prevalence (95% confidence interval) of benefit finding and post-traumatic growth in colorectal cancer survivors

	***N***	**Mean (s.d.)** a	**Prevalence of BF/PTG**	**Prevalence of moderate/high BF/PTG**
BF total	470	3.4 (0.9)	98.9% (97.5–99.7)	64.0% (59.5–68.4)
Acceptance	474	3.6 (1.1)	97.1% (95.1–98.4)	61.6% (57.1–66.0)
Sensitivity to others	464	3.4 (1.1)	97.4% (95.5–98.7)	56.9% (52.2–61.5)
Improved coping	469	3.3 (1.1)	96.2% (94.0–97.7)	56.3% (51.7–60.8)
New purpose of life	479	3.3 (1.2)	93.6% (91.0–95.7)	53.2% (48.6–57.8)
				
PTG total	462	2.0 (1.1)	97.6% (95.8–98.8)	45.9% (41.3–50.6)
Spiritual change	465	1.7 (1.5)	76.3% (72.2–80.1)	32.5% (28.2–36.9)
Appreciation of life	465	2.8 (1.3)	96.1% (94.0–97.7)	70.3% (65.9–74.4)
New possibilities	460	1.6 (1.2)	91.5% (88.6–93.9)	30.0% (25.8–34.4)

Abbreviations: BF=benefit finding; PTG=post-traumatic growth; s.d.=standard deviation.

a The BF scales ranged from 1 to 5, the PTG scales from 0 to 5.

**Table 4:** Determinants of moderate-to-high benefit finding and post-traumatic growth in colorectal cancer survivors

	**Moderate-to-high benefit finding**			**Moderate to high post-traumatic growth**		
	***N* (%)**	**OR_crude_**	**OR_adjusted_** a	***N* (%)**	**OR_crude_**	**OR_adjusted_** a
**Psycho-social factors**						
*Sex*						
Female	117 (67)	1.00 (Ref.)	1.00 (Ref.)	83 (49)	1.00 (Ref.)	1.00 (Ref.)
Male	184 (63)	0.84 (0.57–1.25)	0.87 (0.55–1.37)	129 (44)	0.84 (0.58–1.23)	0.93 (0.61–1.44)
						
*Age at follow-up (years)*						
−59	37 (69)	0.91 (0.44–1.90)	0.95 (0.42–2.15)	31 (57)	1.93 (0.97–3.84)	1.88 (0.88–4.05)
60–69	82 (61)	0.66 (0.37–1.18)	0.62 (0.34–1.15)	64 (48)	1.31 (0.75–2.26)	1.03 (0.70–2.30)
70–79	120 (62)	0.68 (0.40–1.17)	0.63 (0.36–1.12)	82 (43)	1.10 (0.65–1.84)	1.09 (0.63–1.88)
80+	62 (71)	1.00 (Ref.)	1.00 (Ref.)	35 (41)	1.00 (Ref.)	1.00 (Ref.)
		*P*_*trend*_=0.6245	*P*_*trend*_=0.6769		*P*_*trend*_=0.0522	*P*_*trend*_=0.0999
						
*Living with a partner at follow-up*						
No	70 (66)	1.00 (Ref.)	1.00 (Ref.)	197 (55)	1.00 (Ref.)	1.00 (Ref.)
Yes	229 (63)	0.89 (0.56–1.40)	1.07 (0.64–1.80)	162 (45)	0.91 (0.58–1.41)	0.86 (0.52–1.42)
						
*School education* b						
⩽ 9 years	215 (67)	1.00 (Ref.)	1.00 (Ref.)	147 (47)	1.00 (Ref.)	1.00 (Ref.)
10–11 years	53 (65)	0.92 (0.55–1.55)	0.90 (0.52–1.55)	41 (51)	1.15 (0.71–1.88)	1.11 (0.66–1.86)
12+ years	32 (47)	**0.43** **(0.26–0.74)**	**0.45** **(0.25–0.78)**	23 (34)	**0.57** **(0.33–0.99)**	0.59 (0.33–1.06)
		***P*_trend_=0.0046**	***P*_trend_=0.0088**		*P*_*trend*_=0.1194	*P*_*trend*_=0.1523
						
*Number of real friends* b						
0–2	64 (60)	1.00 (Ref.)	1.00 (Ref.)	43 (42)	1.00 (Ref.)	1.00 (Ref.)
3–5	136 (67)	1.33 (0.82–2.17)	1.41 (0.85–2.34)	94 (47)	1.24 (0.77–2.00)	1.25 (0.76–2.07)
6+	101 (63)	1.11 (0.67–1.83)	1.13 (0.67–1.92)	75 (47)	1.25 (0.76–2.06)	1.27 (0.75–2.16)
		*P*_*trend*_=0.8185	*P*_*trend*_=0.7778		*P*_*trend*_=0.4287	*P*_*trend*_=0.4051
						
*Number of comorbidities* b						
0	120 (60)	1.00 (Ref.)	1.00 (Ref.)	89 (45)	1.00 (Ref.)	1.00 (Ref.)
1	102 (68)	1.37 (0.88–2.14)	1.26 (0.80–2.01)	79 (53)	1.40 (0.91–2.14)	1.36 (0.87–2.12)
2+	75 (66)	1.30 (0.80–2.11)	1.13 (0.67–1.88)	43 (40)	0.82 (0.51–1.32)	0.85 (0.51–1.41)
		*P*_*trend*_=0.2184	*P*_*trend*_=0.5523		*P*_*trend*_=0.6463	*P*_*trend*_=0.7522
						
**Clinical factors**						
*Stage* b						
I	94 (61)	1.00 (Ref.)	1.00 (Ref.)	60 (40)	1.00 (Ref.)	1.00 (Ref.)
II	110 (66)	1.21 (0.77–1.91)	1.08 (0.65–1.78)	70 (43)	1.11 (0.70–1.73)	1.03 (0.63–1.69)
III	87 (64)	1.09 (0.68–1.76)	0.91 (0.44–1.88)	74 (54)	1.77 (1.11–2.83)	1.50 (0.75–3.01)
IV	10 (77)	2.09 (0.55–7.92)	1.42 (0.31–6.42)	8 (62)	2.37 (0.74–7.60)	1.79 (0.46–6.88)
		*P*_*trend*_=0.4574	*P*_*trend*_=0.9517		***P*_trend_=0.0089**	*P*_*trend*_=0.2590
						
*Adjuvant therapy*						
None	171 (63)	1.00 (Ref.)	1.00 (Ref.)	109 (41)	1.00 (Ref.)	1.00 (Ref.)
Radiotherapy	6 (50)	0.60 (0.19–1.90)	0.56 (0.16–1.94)	5 (42)	1.04 (0.32–3.35)	1.02 (0.30–3.52)
Chemotherapy	74 (66)	1.16 (0.73–1.84)	1.04 (0.52–2.06)	57 (52)	**1.59** **(1.02–2.49)**	0.99 (0.51–1.90)
Both	50 (69)	1.30 (0.75–2.25)	1.27 (0.63–2.55)	41 (55)	**1.80** **(1.07–3.03)**	1.23 (0.64–2.37)
		*P*_*trend*_=0.3129	*P*_*trend*_=0.5347		***P*_trend_=0.0077**	*P*_*trend*_=0.5872
						
*Colorectal cancer recurrence*						
No	275 (63)	1.00 (Ref.)	1.00 (Ref.)	192 (45)	1.00 (Ref.)	1.00 (Ref.)
Yes	26 (77)	1.90 (0.84–4.30)	1.69 (0.71–4.00)	20 (59)	1.76 (0.86–3.57)	1.55 (0.72–3.33)
						
**Psycho-social factors**						
*Burden of diagnosis* c						
Not at all	22 (63)	0.75 (0.36–1.56)	0.91 (0.41–2.01)	9 (27)	**0.33** **(0.15–0.74)**	**0.36** **(0.15–0.84)**
Little	37 (58)	0.61 (0.35–1.06)	0.57 (0.32–1.04)	24 (38)	**0.54** **(0.31–0.95)**	0.58 (0.32–1.06)
Modest	52 (55)	**0.54** **(0.33–0.87)**	**0.59** **(0.36–0.98)**	36 (38)	**0.54** **(0.33–0.87)**	**0.57** **(0.34–0.95)**
High	185 (69)	1.00 (Ref.)	1.00 (Ref.)	142 (53)	1.00 (Ref.)	1.00 (Ref.)
		*P*_*trend*_=0.0631	*P*_*trend*_=0.1471		***P*_trend_=0.0004**	***P*_trend_=0.0028**
						
*Burden of treatment* c						
Not at all	42 (65)	1.04 (0.58–1.87)	1.25 (0.66–2.38)	22 (35)	**0.54** **(0.30–0.97)**	0.66 (0.35–1.25)
Little	50 (68)	1.19 (0.68–2.10)	1.42 (0.77–2.62)	34 (47)	0.87 (0.51–1.49)	1.01 (0.56–1.80)
Modest	71 (62)	0.92 (0.57–1.48)	1.03 (0.62–1.72)	52 (45)	0.83 (0.51–1.31)	0.93 (0.57–1.52)
High	126 (64)	1.00 (Ref.)	1.00 (Ref.)	99 (50)	1.00 (Ref.)	1.00 (Ref.)
		*P*_*trend*_=0.7070	*P*_*trend*_=0.3114		*P*_*trend*_=0.0605	*P*_*trend*_=0.3155
						
*Identification as cancer patient* c						
No	205 (63)	1.00 (Ref.)	1.00 (Ref.)	145 (45)	1.00 (Ref.)	1.00 (Ref.)
Yes	89 (65)	1.09 (0.72–1.65)	0.94 (0.60–1.48)	60 (45)	0.98 (0.65–1.47)	0.79 (0.51–1.24)
Stress (QSC)c,^d^		1.05 (0.85–1.30)	0.96 (0.76–1.21)		1.02 (0.83–1.24)	0.96 (0.77–1.20)
Depression score (GDS)c,^e^		0.95 (0.90–1.00)	**0.91** **(0.86–0.97)**		**0.93** **(0.88–0.99)**	**0.92** **(0.86–0.98)**

Abbreviations: OR=odds ratio; QSC=questionnaire on stress in cancer patients; GDS=geriatric depression scale. Significant *P*-values (*P*<0.05) and significant associations are hightlighed in bold.

a Adjusted for sex, age at follow-up, living with a partner, education, number of real friends, number of comorbidities, stage, adjuvant therapy, and recurrence, not including the determinant of interest.

b Factor was measured at baseline.

c Factor was measured at the 5-year follow-up.

d ORs refer to an increment of the QSC summary score by 1.

e ORs refer to an increment of the GDS by 1 (i.e., reporting one more depressive symptom).

**Table 5:** Correlation of benefit finding and post-traumatic growth with quality of life in colorectal cancer survivors (Pearson's correlation coefficient *r*)

	**QL**	**PF**	**RF**	**CF**	**EF**	**SF**
*BF total*	0.0537	−0.0264	−0.0008	0.0083	0.0261	0.0038
Acceptance	−0.0054	−0.0505	−0.0561	−0.0568	−0.0190	−0.0753
Sensitivity to others	0.0356	−0.0133	−0.0112	−0.0339	−0.0022	−0.0284
Improved coping	0.0637	−0.0128	0.0192	0.0589	0.0496	0.0388
New purpose of life	0.0885	−0.0019	0.0297	0.0495	0.0433	0.0544
						
*PTG total*	0.1180^*^	0.1001^*^	0.0751	0.0673	0.0334	0.0120
Spiritual change	0.0149	−0.0153	−0.0098	0.0245	−0.0440	−0.0673
Appreciation of life	0.1544^**^	0.1094^*^	0.0913	0.0833	0.0900	0.0602
New possibilities	0.1260^***^	0.1327^***^	0.0951^*^	0.0636	0.0329	0.0276

Abbreviations: QL=overall quality of life; PF=physical functioning; RF=role functioning; CF=cognitive functioning; EF=emotional functioning; SF=social functioning; BF=benefit finding; PTG=post-traumatic growth.

^*^P<0.05;

^**^P<0.001;

^***^P<0.01.

**Table 6:** Mean and standard deviation of quality of life and functioning in colorectal cancer survivors according to the level of total benefit finding and total post-traumatic growth

	**QL**	**PF**	**RF**	**CF**	**EF**	**SF**
*BF*						
Low	63.6 (24.4)	78.9 (23.7)	74.8 (33.2)	78.4 (25.4)	72.9 (26.5)	77.5 (31.6)
High	64.2 (22.8)	75.8 (22.1)	72.0 (29.1)	79.3 (23.1)	74.0 (23.7)	75.4 (28.5)
*P*	0.7918	0.1503	0.3421	0.6865	0.6549	0.4579
*P*_*adj*_^a^	0.6812	0.2276	0.4169	0.8036	0.6647	0.4376
						
*PTG*						
Low	62.1 (24.6)	75.5 (24.3)	71.6 (32.6)	77.9 (25.5)	73.2 (26.3)	76.4 (31.0)
High	66.6 (21.8)	79.1 (20.0)	74.9 (27.9)	80.2 (22.0)	74.1 (22.8)	75.6 (28.1)
*P*	**0.0416**	0.0854	0.2416	0.3000	0.6981	0.7593
*P*_*adj*_^a^	**0.0177**	0.1495	0.2320	0.5693	0.4870	0.9152

Abbreviations: QL=overall quality of life; PF=physical functioning; RF=role functioning; CF=cognitive functioning; EF=emotional functioning; SF=social functioning; BF=benefit finding; PTG=post-traumatic growth.

Significant *P*-values (*P*<0.05) are highlighted in bold.

a Adjusted for all factors that were significant in the analysis of the determinants: education, burden of diagnosis, burden of treatment, therapy, stage, and depression score.
